# The prognosis and incidence of hepatic encephalopathy of patients with liver cirrhosis treated with proton pump inhibitors: A multicenter retrospective study in Japan

**DOI:** 10.1097/MD.0000000000026902

**Published:** 2021-08-13

**Authors:** Akira Sakamaki, Kenya Kamimura, Takeshi Yokoo, Akihiko Osaki, Seiichi Yoshikawa, Yoshihisa Arao, Toru Setsu, Hiroteru Kamimura, Nobuo Waguri, Manabu Takeuchi, Kazuhiro Funakoshi, Shuji Terai

**Affiliations:** a Division of Gastroenterology and Hepatology, Graduate School of Medical and Dental Sciences, Niigata University, Niigata, Niigata, Japan; b Division of Gastroenterology and Hepatology, Niigata City General Hospital, Niigata, Niigata, Japan; c Division of Gastroenterology and Hepatology, Nagaoka Red Cross Hospital, Nagaoka, Niigata, Japan; d Division of Gastroenterology and Hepatology, Niigata Central Prefectural Hospital, Joetsu, Niigata, Japan.

**Keywords:** gastrointestinal bleeding, hepatic encephalopathy, liver cirrhosis, prognosis, proton pump inhibitor

## Abstract

Gastrointestinal bleeding, hepatic encephalopathy (HE), and hepatocarcinogenesis are associated with the prognosis of patients with liver cirrhosis (LC). Proton pump inhibitors (PPIs) have been used to prevent bleeding, however the effects of PPIs on overall survival have not yet been elucidated. Therefore, this multicenter retrospective study aimed to assess the effect of PPI on the prognosis and HE occurrence of the patients with liver cirrhosis in Japan.

A total of 456 patients diagnosed with LC at the 4 institutes during the study period (2010–2014) were assessed. PPI-treated and non-treated patients were compared using propensity score matching analysis. Primary and secondary endpoints of the study were set as the occurrence of HE and overall survival, respectively.

A comparison of all cases showed a significantly poorer hepatic reserve function in the PPI-treated patients. The propensity-score matching analysis was performed and 120 PPI-treated patients were 1:1 matched with non-treated patients. The analysis revealed a higher incidence of HE in the PPI-treated than in the non-treated patients (*P* = .032; hazard ratio [HR], 2.162; 95% confidence interval [CI], 1.066–4.176), but the prognosis of PPI-treated patients was no worse than that of non-treated patients (*P* = .676; HR, 1.101; 95% CI, 0.702–1.726).

This retrospective study showed that PPI administration for the patients with liver cirrhosis may partly be related to the increased incidence of HE but not worsen the patient prognosis.

## 1 Introduction

Liver cirrhosis (LC) is the end-stage of chronic liver disease, and has a median survival time of 33 months. Gastrointestinal (GI) bleeding, hepatic encephalopathy (HE), and hepatocarcinogenesis are the leading causes of poor prognosis in LC patients.^[1]^ Among them, the occurrence of HE is related to several factors, including an aberrance in gut bacteria, such as dysbiosis and small intestine bacterial overgrowth, which could precipitate bacterial translocation;^[2–4]^ and GI bleeding, which triggers the increase of nitrogen compounds due to blood in the gut.^[5]^ Management of GI bleeding is therefore essential.^[6]^ Proton pump inhibitors (PPIs) have been used for treating ulcerative lesions^[7]^ and variceal lesions.^[8,9]^ and have shown efficacy in preventing the recurrence of bleeding. In addition, a recent randomized placebo-controlled trial including 17,598 patients, showed that there was no association between pantoprazole and any adverse events, except for an increased risk of enteric infections, over a three-year period.^[10]^ On the other hand, HE and PPI administration have been reported to be related^[11]^ and Nardelli et al, assessing 310 patients in Italy, reported an association between PPI and minimal HE, overt HE, and mortality.^[12]^ In this study, we assessed 456 patients with liver cirrhosis using propensity score matching to confirm the relation between PPI and HE occurrence and the prognosis in a Japanese cohort.

## 2 Materials and methods

### 2.1 Data collection and inclusion and exclusion criteria

This multicenter retrospective study was performed in the Niigata Prefecture of Japan, and data were collected from 4 hospitals: Nagaoka Red Cross Hospital, Niigata Central Prefectural Hospital, Niigata City General Hospital, and Niigata University Hospital. The study was approved by the ethical review board of Niigata University (Number 2018–0193). We collected data from hospital medical records of patients diagnosed with LC between January 2010 and December 2014 followed by the mean observation period of 3.1 ± 1.4 years. LC diagnosis was based on clinical evidence, such as chronic changes in liver and spleen morphology, demonstrated by imaging, with thrombocytopenia, HE, and esophagogastric varices. Patients with a history of hepatocellular carcinoma (HCC) were included if they showed no recurrence for more than 3 years after the last treatment. The PPI-treated group had PPI administered for more than 6 weeks at the time of inclusion. Patients with secondary liver dysfunction, including those with hepatic congestion and metastatic liver tumors, were excluded. A total of 672 patients satisfied the study criteria, including 111, 130, 177, and 254 patients from Nagaoka Red Cross Hospital, Niigata Central Prefectural Hospital, Niigata City General Hospital, and Niigata University Hospital, respectively.

**Figure 1:**
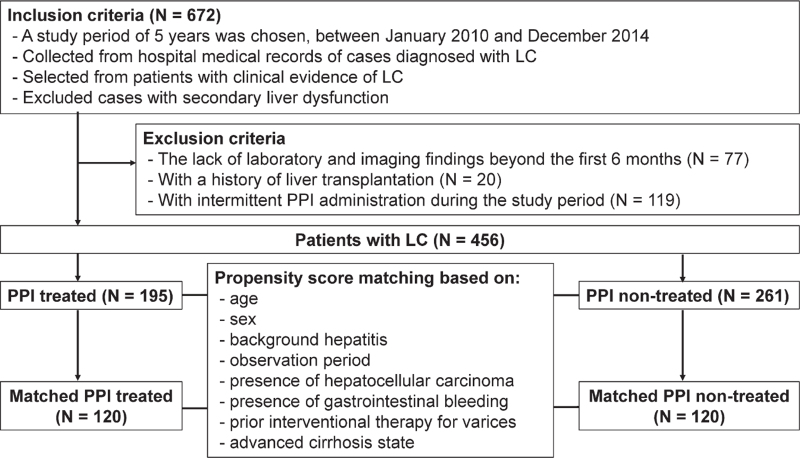
The patient selection process for the multicenter retrospective study to determine the effects of proton pump inhibitors for gastric bleeding on the incidence of hepatic encephalopathy and prognosis in patients with liver cirrhosis. LC = liver cirrhosis, PPI = proton pump inhibitor.

Among the 672 patients, 77 patients who lacked laboratory and/or imaging findings after the first 6 months, 20 patients who had a history of liver transplantation, and 119 patients who had intermittent of PPI administration during the study period were excluded. The remaining 456 patients were assessed in the final analyses and PPI-treated and non-treated groups were defined as patients who were and were not treated with PPIs for the entire study period, respectively. Fig. 1 summarizes the patient selection process. The primary study endpoint was set as the incidence of a new occurrence of HE during the observation period, and the secondary study endpoint was set as overall survival. Onset of HE was defined as overt HE symptoms (≥grade 2)^[13]^ with treatment intervention including intravenous branched-chain amino acid infusions.

### 2.2 Statistical analysis

The PPI-treated and non-treated patient groups were compared using propensity-score matching analysis with adjusting factors including age; sex; background hepatitis (alcoholic hepatitis or viral and alcoholic combined hepatitis); observation period; presence of HCC; presence of GI bleeding (meaning the events of GI bleeding from the gastroesophageal varices, gastroduodenal ulcers, Mallory-Weiss syndrome, and angiodysplasia over the entire study period); prior interventional therapy for varices, including endoscopic injection sclerotherapy (EVL), balloon-occluded retrograde transvenous obliteration (BRTO), and surgery; advanced cirrhosis state (a Child–Pugh score of ≥7 or treatment for complications of LC, including loop diuretics, branched-chain amino acids, synthetic disaccharides, and poorly absorbable oral antibiotics). The Kolmogorov–Smirnov test was used to assess the normality of the distribution of continuous variables. Wilcoxon signed-rank sum and Fisher exact tests were used to compare data at the study entry point, and the cumulative incidence plots method with the log-rank test was used to compare HE, GI bleeding, and prognosis. SPSS Statistics software (version 22.0; IBM, Armonk, NY, USA) was used to perform the Kolmogorov–Smirnov test, Mann–Whitney *U* test, Wilcoxon signed-rank sum test, and propensity score matching, whereas Prism Software (version 6.07, 2015; GraphPad, La Jolla, CA, USA) was used to perform the log-rank test and cumulative incidence plots.

## 3 Results

The study cohort included 279 males and 177 females, with an average age of 66.8 ± 11.3 years. PPI treatment was used in 195 patients (42.8%). Table 1 shows the rate of PPIs. Rabeprazole was the most frequently used PPI (51.3%), followed by lansoprazole, omeprazole, and esomeprazole. The mean observation period in the entire cohort was 3.1 ± 1.4 years. Furthermore, 63 patients (13.8%) had HE as a complication, and 122 (26.8%) died during the study period. A simple comparison of PPI-treated and non-treated patients showed statistical differences in: age (younger in PPI-treated, *P* < .001), Child-Pugh scores (higher in PPI-treated, *P* = .001), model for end-stage liver disease scores (higher in PPI-treated, *P* = .005), onset of HE (higher in PPI-treated, *P* = .017), and onset of GI bleeding (higher in PPI-treated, *P* < .001), however there was no difference in hepatocarcinogenesis (*P* = .523) (Table 2). To further analyze the actual effects of PPI on the prevention of HE occurrence, the 2 groups were compared using propensity score matching analysis after adjusting for factors including age, sex, background hepatitis, observation period, presence of HCC, presence of GI bleeding, prior history of interventional therapy for varices, and advanced cirrhosis state (see materials and methods section for the details). One hundred twenty PPI treated and non-treated patients were 1:1 matched and no significant differences were found between the 2 groups in terms of age, sex, background hepatitis, liver function, kidney function, and complication of HCC and GI bleeding (Table 3).

**Table 1:** PPIs used in the cases in this study.

PPI	n (%)
Rabeprazole	100 (51.3)
Lansoprazole	52 (26.7)
Omeprazole	15 (7.7)
Esomeprazole	11 (5.6)
Several types	17 (8.7)

**Table 2:** PPI treated and non-treated without a propensity score matching analysis.

Groups	PPI treated N = 195	PPI non-treated N = 261	*P* value by Mann–Whitney *U* or Fisher exact test
Age, yrs (mean ± SD)	64.6 ± 11.1	68.4 ± 11.1	<.001
Gender, n (%)			.654
Males	117 (60.0)	162 (62.1)	
Females	78 (40.0)	99 (37.9)	
Background hepatitis, n (%)			.056
Hepatitis B	18 (9.2)	21 (8.0)	
Hepatitis C	58 (29.7)	110 (42.1)	
Alcoholic hepatitis	69 (35.4)	73 (28.0)	
Others	50 (25.7)	57 (21.8)	
Total bilirubin, mg/dL	1.6 ± 1.7	1.4 ± 1.9	<.001
Prothrombin time, %	73.6 ± 18.2	81.4 ± 19.2	<.001
Albumin, g/dL	3.5 ± 0.6	3.7 ± 0.6	.001
Creatinine, mg/dL	0.96 ± 1.09	0.92 ± 0.79	.512
Child-Pugh Score	6.8 ± 1.8	6.2 ± 1.4	.001
MELD score	7.2 ± 5.3	5.9 ± 4.6	.005
Agents for LC complications, n (%)	147 (75.4)	137 (52.5)	<.001
Loop diuretics, n (%)	105 (53.8)	94 (36.0)	<.001
Synthetic disaccharides, n (%)	38 (19.5)	42 (16.1)	.346
Oral poorly absorbable antibiotics, n (%)	18 (9.2)	7 (2.7)	.002
LC statement, n (%)			<.001
Decompensated	164 (84.1)	174 (66.7)	
Compensated	31 (15.9)	87 (33.3)	
5-year incidence rate of HCC, %	51.1	56.4	.523
5-year incidence rate of HE, %	24.8	14.8	.017
5-year incidence rate of GI bleeding, %	17.9	4.6	<.001
Observation period, years (mean ± SD)	3.1 ± 1.4	3.2 ± 1.4	.327
5-year survival rate, %	55.6	62.3	.228

**Table 3:** PPI treated and non-treated using a propensity score matching analysis with adjusted factors.

Groups	PPI treated N = 120	PPI non-treated N = 120	*P* value by Wilcoxon signed-rank sum or Fisher exact test
Age, yrs (mean ± SD)	67.5 ± 10.3	68.4 ± 10.9	.542
Gender, n (%)			1.000
Males	73 (60.8)	73 (60.8)	
Females	47 (39.2)	47 (39.2)	
Background hepatitis, n (%)			.313
Hepatitis B	11 (9.2)	8 (6.7)	
Hepatitis C	32 (26.7)	45 (37.5)	
Alcoholic hepatitis	43 (35.8)	40 (33.3)	
Others	34 (28.3)	27 (22.5)	
Total bilirubin, mg/dL	1.5 ± 1.8	1.4 ± 1.5	.184
Prothrombin time, %	75.1 ± 18.8	79.1 ± 21.2	.118
Albumin, g/dL	3.6 ± 0.7	3.6 ± 0.7	.317
Creatinine, mg/dL	1.06 ± 1.31	1.02 ± 1.11	.911
Child-Pugh Score	6.5 ± 1.7	6.5 ± 1.5	.912
MELD score	7.6 ± 5.8	6.5 ± 5.1	.073
Agents for LC complications, n (%)	89 (74.2)	77 (64.2)	.093
Loop diuretics, n (%)	62 (51.7)	55 (45.8)	.366
Synthetic disaccharides, n (%)	23 (19.2)	28 (23.3)	.430
Oral poorly absorbable antibiotics, n (%)	10 (8.3)	6 (5.0)	.301
LC statement, n (%)			.739
Decompensated	97 (80.8)	99 (82.5)	
Compensated	23 (19.2)	21 (17.5)	
5-year incidence rate of HCC, %	48.5	56.8	.840
5-year incidence rate of HE, %	25.1	12.3	.032
5-year incidence rate of GI bleeding, %	7.7	10.1	.721
Observation period, yrs (mean ± SD)	3.0 ± 1.4	3.1 ± 1.4	.640
5-year survival rate, %	52.4	54.5	.676

**Figure 2:**
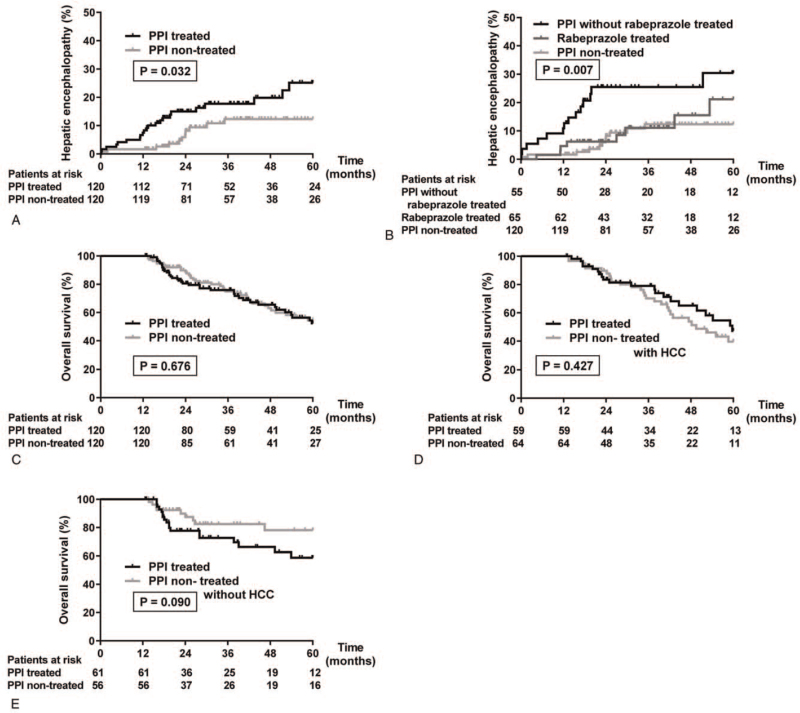
Cumulative incidence plots for PPI treated and non-treated patients using propensity-score matching analysis with adjusted factors. (A) The incidence of hepatic encephalopathy among the PPI-treated and non-treated patients. (B) Effect of rabeprazole on hepatic encephalopathy. (C) Overall survival of liver cirrhosis patients with or without PPI treatment. (D, E) After dividing patients according to the presence of HCC, there was no difference in the prognoses of patients with (D) and without (E) HCC among either the PPI-treated or the PPI non-treated groups. PPI = proton pump inhibitor, HCC = hepatocellular carcinoma.

The incidence of HE was higher among PPI-treated than PPI non-treated patients (*P* = .032; hazard ratio [HR], 2.162; 95% confidence interval [CI], 1.066–4.176; Fig. 2a). In addition, in subgroup analyses among the PPI-treated patients, rabeprazole was associated with fewer HE cases than other PPIs (3 groups, *P* = .007; rabeprazole vs other PPIs, *P* = .060; rabeprazole vs PPI non-treated, *P* = .493; Fig. 2b). Interestingly, although the HE increased in the PPI-treated group in our study cohort, PPIs were not associated with worsen prognoses (five-year survival rate, 52.4% vs 54.5%; *P* = .676; HR, 1.101; 95% CI, 0.702–1.726; Fig. 2c). Patients were divided according to the presence of HCC to investigate the relationship between PPIs and prognosis. The prognoses of LC patients both with and without HCC were no worse in the PPI-treated group than in the non-treated group (with HCC: *P* = .427; HR, 0.796; 95% CI, 0.455–1.395; Fig, 2d, without HCC: *P* = .090; HR, 1.968; 95% CI, 0.905–4.093; Fig. 2e).

## 4 Discussion

Our multi-center retrospective study has shown that there is a potential risk of increased HE occurrence associated with frequent use of PPIs and these should therefore only be administered with careful consideration of their necessity on a case-by-case basis, especially for the preventive use.

It has been reported that PPIs may facilitate oral bacterial translocation into the small intestine by suppressing gastric acid production,^[14]^ and worsening dysbiosis via small intestine bacterial overgrowth, thereby increasing the incidence of HE. Comparison of microbiota in PPI-treated and non-treated patients indicated that the change caused by PPIs is similar to that caused by LC progression.^[15]^ PPIs may therefore progress liver dysfunction by changing gut microbiota. Few reports have shown the clinical association between PPIs and HE and the results regarding this association have been inconsistent **(**Table 4**)**.^[16–24]^ Meta-analysis of these reports appears to demonstrate a positive relationship indicating that PPI-treatment may increase the risk of HE.^[25–27]^ Furthermore, other reports comparing LC patients with and without HE concluded that PPI administration is an independent factor associated with HE.^[28,29]^ Conversely, several reports suggest that it is premature to decide whether PPIs induce the development of HE in cirrhotic patients^[11,12]^ because of the difference in background adjustment. In our cohort, the backgrounds of PPI-treated and non-treated patients were significantly different in terms of liver function and onset of GI bleeding. In fact, the PPI-treated group in our study showed poorer hepatic functions (evidenced by worse Child-Pugh scores and MELD scores) and had higher chances of GI bleeding events treated with PPIs. Therefore, a propensity score matching analysis was performed to aid in these comparisons and to determine the effects of PPI on HE and prognosis. Based on these meticulous analyses, the associations between the type of PPIs and HE in our findings were similar to those of previous reports.^[20,22]^ Our results further implied an association between PPIs and the levels in hepatocytes of cytochrome P450, which delays metabolism of certain PPIs in patients with LC.^[30–34]^ Both HE (2) and PPI-treatment^[35]^ have been reported to be independent risk factors for mortality in patients with LC, however this might be related to the type of PPI administered.^[22]^ Although some reports indicate no significant associations between PPI administration and LC prognosis,^[36]^ it is clear, that complications of HE and GI bleeding are associated with worse prognoses in LC patients. By matching the factors of GI bleeding, liver function, and HCC incidence, our study demonstrated that similar to these previous reports PPI administration increased the risk of HE occurrence.^[25–27]^ Interestingly however, we observed no effect of PPI administration on patient's prognoses as de Vos M, et al reported.^[36]^ This study demonstrated for the first time that long-term PPI administration is related to a higher incidence of HE, but did not affect the overall prognosis of a Japanese patient cohort. Notably, a systematic review has also indicated that the long term administration of PPIs does not prevent GI bleeding;^[37]^ therefore, it is important to assess the benefit of PPIs for patients with LC on a case-by-case basis, taking the endoscopic findings in GI, bleeding tendency, alcohol abuse, and other factors into consideration.

**Table 4:** Summary of the reported studies.

Author	Year	Study type	Number of cases (PPI treated / non-treated)	Observation period	Age (average) (PPI treated / non-treated)	Men (%) (PPI treated / non-treated)	Decompensated LC (%) (PPI treated / non-treated)	GI bleeding (%) (PPI treated / non-treated)	HCC (%) (PPI treated / non-treated)	List of PPIs	Effect of PPI on HE
Terg K^17^	2015	Prospective	165 / 219	3 mo	57 / 57	68 / 77	100 / 100	Excluded	NA	NA	Negative
Dam G^18^	2016	Retrospective	340 / 525	30 mo	58 / 57	68 / 69	100 / 100	NA	NA	NA	Positive
Huang KW^19^	2016	Retrospective	1870 / 1190	13 yrs	54 / 53∗	73 / 81∗	100 / 100	63 / 27∗	NA	NA	Negative
Cole HL20	2016	Retrospective	114 / 92	2 yrs	57 / 56	74 / 55∗	100 / 100	30 / 15∗	NA	NA	Positive
Tsai CF^21^	2017	Retrospective	2332	13 yrs	53	74	NA	NA	NA	Lansoprazole, 29% Rabeprazole, 8%	Positive
Schiavon LL^22^	2017	Prospective	93 / 98	32 mo	57 / 52∗	63 / 73	100 / 100	62 / 43∗	Excluded	NA	Positive
Hung TH^23^	2018	Retrospective	1004 / 4016	1 yrs	62 / 63	68 / 67	NA	Excluded	45 / 45	Lansoprazole, 35% Rabeprazole, 9%	Positive
Fasullo M^24^	2019	Retrospective	75/28	NA	60/55	64/47	100/100	31/36	NA	NA	Positive
Nardelli S^25^	2019	Prospective	185/125	NA	63/62	74/67	100/100	37/28	NA	NA	Positive

This study had some limitations. Firstly, factors that directly induced HE including the hypovolemia, constipation, and administration of benzodiazepines^[6]^ and prior history of HE was not assessed, although there were no significant differences between the groups regarding the oral administration of poorly absorbable antibiotics. Secondly, bias with case selection could not be excluded as the onset of GI bleeding was higher in PPI-treated patients and this could be a precipitator for development of HE. Data were gathered from patients with clinically diagnosed LC, and the frequency of the compensated LC cases was lower than that of the decompensated cases. Therefore, distinguishing chronic hepatitis from LC was difficult, and some LC cases might have been excluded. In addition, the difference of the prognosis of LC between ethnics^[38]^ and the pharmacokinetic drug interaction with the activity of PPI needs to be considered in the future study.^[39]^

In conclusion, this multicenter retrospective study showed that PPI treatment might be associated with increased HE occurrence but did not worsen the prognosis of PPI-treated patients with LC (Graphical abstract). Considering the relatively low preventive effect of PPI on GI bleeding in liver cirrhotic cases, it is recommended that PPIs should only be administered with careful consideration. Further research is needed including the development of precision medicine based on clinical information, analysis of the differences between PPIs, and investigation into the potential role of intestinal flora in determining which patients will benefit from treatment.

## Acknowledgments

We would like to thank all the gastroenterologists and hepatologists at Nagaoka Red Cross Hospital, Niigata Central Prefectural Hospital, Niigata City General Hospital, and Niigata University Hospital. They also thank Enago for the critical reading of the manuscript and English language review.

## Author contributions

**Conceptualization:** Akira Sakamaki, Kenya Kamimura.

**Data curation:** Akira Sakamaki, Takeshi Yokoo, Akihiko Osaki, Seiichi Yoshikawa, Yoshihisa Arao, Toru Setsu, Hiroteru Kamimura, Nobuo Waguri, Manabu Takeuchi, Kazuhiro Funakoshi.

**Formal analysis:** Akira Sakamaki, Kenya Kamimura, Takeshi Yokoo, Akihiko Osaki, Seiichi Yoshikawa, Yoshihisa Arao, Toru Setsu, Hiroteru Kamimura, Nobuo Waguri, Manabu Takeuchi, Kazuhiro Funakoshi, Shuji Terai.

**Supervision:** Shuji Terai.

**Validation:** Toru Setsu, Shuji Terai.

**Writing – original draft:** Akira Sakamaki, Kenya Kamimura.

**Writing – review & editing:** Akira Sakamaki, Kenya Kamimura, Takeshi Yokoo, Akihiko Osaki, Yoshihisa Arao, Toru Setsu, Hiroteru Kamimura, Shuji Terai.
